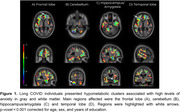# Association between brain metabolism and anxiety in long COVID in an underrepresented cohort

**DOI:** 10.1002/alz.094142

**Published:** 2025-01-09

**Authors:** Débora Guerini de Souza, Maiele Dornelles Silveira, Wyllians Vendramini Borelli, Joana Emilia Senger, Luiza Santos Machado, João Pedro Ferrari‐Souza, Marco Antônio de Bastiani, Guilherme Povala, Ana Paula Bornes da Silva, Guilherme Bastos de Mello, João Pedro Uglione da Ros, Arthur Viana Jotz, Matheus Fakhri Kadan, Graciane Radaelli, Daniele de Paula de Paula Faria, Artur Martins Coutinho, Mychael V Lourenco, Tharick A. Pascoal, Pedro Rosa‐Neto, Cristina Sebastião Matushita, Ricardo Benardi Soder, Artur Francisco Schumacher‐Schuh, Diogo O. Souza, Jaderson Costa da Costa, Eduardo R. Zimmer

**Affiliations:** ^1^ Universidade Federal do Rio Grande do Sul, Porto Alegre, Rio Grande do Sul Brazil; ^2^ Memory Center, Hospital Moinhos de Vento, Porto Alegre, RS Brazil; ^3^ Federal University of Rio Grande do Sul, Porto Alegre, Rio Grande do Sul Brazil; ^4^ University of Pittsburgh, Pittsburgh, PA USA; ^5^ Federal University of Rio Grande do Sul, Brazil, Porto Alegre, RS Brazil; ^6^ Pontifícia Universidade Católica do Rio Grande do Sul, Porto Alegre, Rio Grande do Sul Brazil; ^7^ Lutheran University of Brazil, Canoas, Rio Grande do Sul Brazil; ^8^ Federal University of Rio Grande do Sul, Porto Alegre, RS Brazil; ^9^ Brain Institute, RS, Porto Alegre, Rio Grande do Sul Brazil; ^10^ University of São Paulo Medical School, São Paulo, São Paulo Brazil; ^11^ Universidade Federal do Rio de Janeiro, Rio de Janeiro Brazil; ^12^ Translational Neuroimaging Laboratory, The McGill University Research Centre for Studies in Aging, Montréal, QC Canada; ^13^ Brain Intitute, RS, Porto Alegre, Rio Grande do Sul Brazil; ^14^ Clinical Hospital of Porto Alegre, Porto Alegre, Rio Grande do Sul Brazil; ^15^ Universidade Federal do Rio Grande do Sul, Porto Alegre Brazil; ^16^ Brain Institute of Rio Grande do Sul ‐ Pontifícia Universidade Católica do Rio Grande do Sul, Porto Alegre, Rio Grande do Sul Brazil

## Abstract

**Background:**

Long COVID is an under‐characterized disorder that affects a wide range of individuals after COVID‐19 resolution. Long COVID individuals report persistent neurological manifestations, such as anxiety. Understanding its effects in the brain might help uncover the actual burden imposed by the pandemic sequelae and either define or discard long COVID as a risk factor for neurodegenerative diseases. Here, we aim to identify whether there is an association between brain metabolism and anxiety in an underrepresented population.

**Method:**

Community‐dwelling individuals, above 50 years old, from Porto Alegre, Brazil, were divided into long COVID (n = 39) and control groups (n = 10) were evaluated with a battery of neuropsychological testing, including the GAD‐7 scale of anxiety. Then, they underwent a brain [18F]FDG‐PET scan (images normalized by the pons). We conducted a voxel‐wise linear regression testing the association between [18F]FDG metabolism and GAD‐7, and corrected for education, sex, and age. The analysis was corrected for multiple comparisons using the cluster‐wise random field theory method (significant t←3.34 and t>3.34, p<0.001, df = 35).

**Result:**

We found that GAD‐7 score presented a widespread negative association with [18F]FDG metabolism in multiple gray and white matter regions (Figure 1). Specifically, hippocampus (tmax = ‐3.34, p = 0.002), amygdala (tmax = ‐3.82, p = 0.0005), cerebellum (tmax = ‐4.28, p = 0.0001), and lateral occipitotemporal gyrus (tmax = ‐5.26, p = 0.0001) had the most relevant associated clusters in gray matter, while temporal lobe (tmax = ‐3.9, p = 0.0004) and frontal lobe (tmax = ‐4.33, p = 0.0001) presented the most relevant associated clusters in white matter.

**Conclusion:**

Anxiety symptoms are a highly self‐reported symptom in long COVID. Here we show that anxiety is widely associated with reduced brain glucose metabolism in crucial areas for the limbic system and cognition, such as the hippocampus and amygdala. The peculiar associations between anxiety and FDG metabolism in white matter may suggest inflammatory responses triggered by long COVID. These data provide new insights into the mechanisms underlying long COVID symptoms in the brain.